# “WhatsBarb” Citizen Surveillance: Survey of Insects Mistaken for Triatomines

**DOI:** 10.1590/0037-8682-0240-2024

**Published:** 2025-03-31

**Authors:** Matheus de Araújo Paz, Anne Caroline Alves Meireles, Cleber Galvão, Hélcio Reinaldo Gil-Santana, Genimar Rebouças Julião

**Affiliations:** 1 Fundação Oswaldo Cruz Rondônia, Laboratório de Entomologia, Porto Velho, RO, Brasil.; 2 Fundação Oswaldo Cruz, Instituto Oswaldo Cruz, Laboratório Nacional e Internacional de Referência em Taxonomia de Triatomíneos, Rio de Janeiro, RJ, Brasil.; 3 Fundação Oswaldo Cruz, Instituto Oswaldo Cruz, Laboratório de Diptera, Rio de Janeiro, RJ, Brasil.; 4 Fundação Oswaldo Cruz Rondônia, Instituto Nacional de Ciência e Tecnologia de Epidemiologia da Amazônia Ocidental, Porto Velho, RO, Brasil.

**Keywords:** biodiversity, Chagas disease, digital health, heteropteran, volunteers

## Abstract

**Background:**

Triatomine recognition by the public is an important factor in Chagas disease (CD) prevention campaigns. Citizen surveillance has been demonstrated as an effective alternative strategy, increasing the possibility of monitoring vector populations on a broad geographic scale. Thus, our study aimed to explore a database built from pictorial/video records and specimens sent through citizen participation, identify the main groups confused with triatomines, and systematize the data on insect diversity.

**Methods:**

Due to demand from the local community, the “WhatsBarb” project was established using a digital platform application, to receive photos of suspected insects, or triatomines, and provide digital campaigns, with instructions for collecting insects and delivery to Entomological Surveillance Services.

**Results:**

In total, 465 insect records were obtained between 2019 and 2024. We identified taxonomic orders and families in 464 and 454 records, respectively, and estimated at least 68 genera, 42 species, and 101 distinct taxa. Triatomines represented 32.3% of the volunteer contacts. Records showed a predominance of true bugs (Hemiptera: Heteroptera), including phytophagous species and predator reduviids (54.6%; n=254). Contacts were received from 20 of the 26 states in Brazil, in addition to the Federal District.

**Conclusions:**

Our findings demonstrate the power of digital tools in public health, which could aid in describing, preventing, and controlling CD vectors, and could be applied to other neglected diseases.

## INTRODUCTION

Chagas disease (CD), also known as American Trypanosomiasis (AT), is a neglected disease that has gained prominence due to its globalization and is currently occurring in countries where it was not previously reported^1^ ^,^ ^2^. In northern Brazil, oral transmission is the main form of contamination and is responsible for the majority of cases of acute Chagas disease (ACD). In turn, the number of cases due to vector classical transmission has decreased, with no records of domiciliated triatomine species in this region^1^ ^,^ ^2^.

In Brazil, 4,019 confirmed cases of ACD were recorded between 2006 and 2022, with the vast majority of infections caused by *Trypanosoma cruzi* occurring in the Amazonian states (northern region) (n=3,800, 94.5%). However, only seven cases of autochthonous ACD were registered in the state of Rondônia during the same period^3^. This low number suggests gaps in the epidemiological history of ACD in this Amazonian state, which is characterized by several migratory waves and environmental changes^4^ ^,^ ^5^.

The Brazilian program for CD surveillance program is based on passive surveillance with community participation. Considering that the main modes of transmission of CD - oral and vector direct contamination^2^ ^,^ ^6^ - are mediated by triatomines, insect recognition by the general public is essential. Several insect orders, taxa, and even arachnids cause uncertainty among the population who attribute the diagnosis to kissing bugs or triatomines, that is, the vector of *Trypanosoma cruzi,* the causal agent of CD^7^ ^-^ ^9^.

Citizen surveillance has been demonstrated to be an effective strategy for epidemiological monitoring systems, increasing the speed of information dissemination and possibility of monitoring vector populations on a broad geographic scale^10^ ^-^ ^14^. 

Furthermore, pictorial databases such as iNaturalist have been widely used as sources of scientific data^15^, allowing an understanding of the factors that affect the distribution of arthropod vectors in time and space^16^, in addition to the recording of rare species^17^. Despite these advantages, databases may only have partial applications considering the taxonomic identity of organisms. Despite advances in digital technologies, specialist collaborations are still necessary for correct diagnosis^18^. Thus, digital tools resulting from the combination of professional expertise and artificial intelligence can increasingly contribute to surveillance, with the participation of society presenting low cost, speed, and an unlimited geographic scale^19^. 

Considering the decline in global insect populations and some specific groups^20^, the multimedia platform WhatsApp can be used to expand dialogue with the population and collect information about biodiversity quickly and cheaply, in addition to improving prevention efforts for endemic and neglected diseases. Due to the population's need for insect and triatomine diagnosis and to answer questions about CD, the Fiocruz Rondônia Entomology Laboratory started the “WhatsBarb” project in December 2018. As an alternative strategy for public health surveillance, a contact channel was created through a telephone number on a pre-existing platform application to disseminate mass communication campaigns on triatomines, disease risks, instructions for insect collection, and delivery to surveillance systems.

Thus, our hypothesis-driven taxonomic study aimed to identify the main families and orders confused with triatomines (Hemiptera: Reduviidae) and inventory insect diversity on a local/regional scale based on pictorial/video records and specimens gathered by WhatsBarb volunteer participants. 

## METHODS

### Ethical considerations

Initially, the “WhatsBarb” Project was designed to meet social needs (2019-2022). Participants were asked to adhere to the project, and provided with the Digital Informed Consent Form (TCLE-d) and the “Authorization for the Use of Insect Images.” Volunteers who declared that they were over 18 years of age, (i) agreed to participate in the WhatsBarb project, and (ii) gave authorization for the use of their insect image or video on a digital platform were considered eligible. The Human Research Ethics Committee (CEP-CEPEM) approved this study (CAAE 72372123.1.0000.0011). Data analyses were performed using an anonymous dataset of all the participants (n=465). We selected photographs sent by volunteers who provided consent and authorization (n=67).

### Data Storage and Security Measures

To guarantee volunteer confidentiality and privacy, the data obtained through WhatsBarb had restricted access. Only researchers and students from Fiocruz Rondônia were directly linked to the project. Triatomine images and data were sent to Public Health Surveillance systems such as the Municipal Health Departments, State Coordination of Disease Surveillance Chagas, Entomology Center at LACEN-RO (Central Laboratory of Rondônia), and Vector Control Division/Zoonosis and Entomology Research and Diagnostics Division from SEMUSA Porto Velho for intervention and vector control.

### Diagnosis and Taxonomy of Insects

Our biologist team coded and organized the insect photos according to the taxonomic orders. Digital tools were used, including Google Lens and Google Images, formal literature and taxonomic sites, “Insetos of Brasil”^21^, “Vetores da doença de Chagas no Brasil”^1^, and the Taxonomic Catalog of the Fauna of Brazil (http://fauna.jbrj.gov.br/). After the initial diagnosis, experts were consulted to confirm the identification at the most detailed level possible (Galvão and Gil-Santana). The physical specimens collected or delivered by the participants were pinned by the research team at the Laboratory of Entomology of the Fiocruz Rondônia, identified, labeled with a voucher code, and stored in the Entomological Collection (COLRO).

### Database and Images

The WhatsBarb database was composed of telephone contact and name of the participant; date of contact, in the case of triatomines; notification form number; record of photo sending; photo code; type of insect, whether alive or dead; date of identification; and, in the case of physical specimens, institution of delivery, date of collection, estimated time, and period. Municipality, coordinates, state, type of property, address, insect encounter location, taxonomy (order, sub-order, family, subfamily, genus, and species), stage of development, and sex were also recorded. The images sent by the volunteers were stored on a computer under code names (assigned by the database) and organized into folders. Image publication was dependent on the participants’ authorization.

### Data analysis

Descriptive statistics and exploratory analyses were limited to the geographical range of insect records, year/month, insect survival (dead or alive during the encounter), developmental stage of insects, and taxonomic diagnosis.

## RESULTS

A total of 465 insect records were obtained between 2019 and 2024, primarily in February 2019. Most of the images and specimen records were from adult insects (98.3%). An unidentified egg and seven nymphs were recorded by project volunteers. In January 2019, when the WhatsBarb project began, four Brazilian states were recorded, totaling 28 volunteer participants, of which 25 were from Rondônia. In February 2019, the month with the highest number of contacts, nine states totaled 82 volunteer inquiries, of which 68 were from Rondônia. Three hundred and twenty insects were alive, and 145 were dead at the time of the encounter (Table 1).

**TABLE 1:** Number of insect records obtained through the WhatsBarb digital platform by the Brazilian federative unit, insect general category, and survival status.

States	Total of records	Non-triatomines	Triatomines	Alive	Dead
Acre	2	0	2	2	0
Amazonas	2	2	0	1	1
Bahia	11	11	0	5	6
Ceará	4	3	1	3	1
Distrito Federal	4	4	0	2	2
Goiás	2	2	0	1	1
Maranhão	6	5	1	6	0
Mato Grosso	10	10	0	10	0
Minas Gerais	4	4	0	4	0
Pará	2	1	1	0	2
Paraíba	3	3	0	1	2
Paraná	16	16	0	16	0
Pernambuco	9	9	0	4	5
Piauí	3	3	0	2	1
Rio de Janeiro	15	15	0	14	1
Rio Grande do Norte	2	0	2	1	1
Rio Grande do Sul	6	6	0	6	0
Rondônia	337	194	143	221	116
Santa Catarina	6	6	0	6	0
São Paulo	19	19	0	15	4
Sergipe	1	1	0	0	1
Unknown Location	1	1	0	0	1
**Total**	**465**	**315**	**150**	**320**	**145**

### Taxonomical Diagnosis of the Insects

Using image and specimen analyses, we identified the taxonomic orders and families of 464 and 454 records, respectively, and estimated at least 68 genera, 42 species, and 101 distinct taxa (Supplementary Table 1). There was a predominance of image recordings of true bugs, including phytophagous species and the predator reduviids (54.6%, n=254), with triatomines representing 32.3% of the contacts (n=150). Eight kissing bug species were identified: *Eratyrus mucronatus*, *Panstrongylus geniculatus*, *Panstrongylus lignarius*, *Panstrongylus lutzi*, *Triatoma brasiliensis brasiliensis*, *Rhodnius pictipes*, *Rhodnius* cf. *stali*, and *Rhodnius robustus*, with the latter being the most abundant. Owing to the loss of structures of taxonomic importance, we were unable to identify 20 triatomines and determine the sex of 14 specimens (Table 2). 

**SUPPLEMENTARY TABLE 1:** Taxonomical diagnosis of insect records (images and specimens) obtained from “WhatsBarb” project volunteers, between 2019 and 2024. *Unidentified: Insect was not identified due to image quality.

Order	Family	Genus	Species	Total
**Coleoptera**	**Anthribidae**	*Ptychoderes*	*Ptychoderes* sp.	1
	**Brentidae**	Unidentified*	Unidentified*	1
	**Carabidae**	*Laemostenus*	*Laemostenus* sp.	1
		Unidentified*	Unidentified*	1
	**Cerambycidae**	*Acanthoderes*	*Acanthoderes* spp.	2
		*Achryson*	*Achryson* cf. *surinamum*	1
		*Aegomorphus*	*Aegomorphus* cf. *jaspideus*	3
			*Aegomorphus* spp.	2
		*Dryoctenes*	*Dryoctenes* cf. *scrupulosus*	1
		*Hedypathes*	*Hedypathes* sp.	1
		*Hypselotropis*	*Hypselotropis* sp.	1
		*Macrodontia*	*Macrodontia cervicornis*	1
		*Megaderus*	*Megaderus* cf. *stigma*	1
		*Orthomegas*	*Orthomegas* spp.	2
		*Susuacanga*	*Susuacanga* cf. *octoguttata*	1
		*Taeniotes*	*Taeniotes* sp.	1
		*Trachyderes*	*Trachyderes* spp.	2
	**Curculionidae**	*Amerrhinus*	*Amerrhinus ynca*	1
		*Heilipodus*	*Heilipodus* sp.	1
		*Homalinotus*	*Homalinotus* cf*. humeralis*	1
			*Homalinotus* cf. *coriaceus*	1
			*Homalinotus* sp.	1
		*Metamasius*	*Metamasius* cf. *hemipterus*	4
			*Metamasius* sp.	1
		*Derelomus*	*Derelomus* sp.	1
		*Pissodes*	*Pissodes* sp.	1
		Unidentified*	Unidentified*	2
	**Scarabaeidae**	*Strategus*	*Strategus* sp.	1
	**Staphylinidae**	*Paederus*	*Paederus* sp.	1
	**Tenebrionidae**	Unidentified*	Unidentified*	4
	Unidentified*	Unidentified*	Unidentified*	4
Diptera	**Fanniidae**	Unidentified*	Unidentified*	1
	**Tabanidae**	Unidentified*	Unidentified*	2
	**Tipulidae**	Unidentified*	Unidentified*	2
Hemiptera	Acanthosomatidae	Unidentified*	Unidentified*	5
	**Alydidae**	*Hyalymenus*	*Hyalymenus* sp.	1
		*Neomegalotomus*	*Neomegalotomus* sp.	1
	**Aradidae**	*Dysodius*	*Dysodius* spp.	2
			*Dysodius lunatus*	2
		Unidentified*	Unidentified*	9
	**Belostomatidae**	Unidentified*	Unidentified*	5
	**Coreidae**	*Acanthocephala*	*Acanthocephala* spp.	2
		*Anasa*	*Anasa* spp.	4
		*Cebrenis*	*Cebrenis* cf. *cauta*	1
		*Crinocerus*	*Crinocerus sanctus*	1
		*Hypselonotus*	*Hypselonotus* sp.	1
		*Leptoglossus*	*Leptoglossus* spp.	16
		Unidentified*	Unidentified*	15
		*Pachylis*	*Pachylis laticornis*	3
			*Pachylis* sp.	1
		*Petalops*	*Petalops* cf. *azureus*	1
		*Phthiacnemia*	*Phthiacnemia* cf. *picta*	1
		*Spartocera*	*Spartocera* spp.	92
	**Largidae**	*Rosaphe*	*Rosaphe* cf. *stylophthalmum*	1
	**Miridae**	Unidentified*	Unidentified*	
	**Pentatomida**e	*Edessa*	*Edessa* sp.	1
		Unidentified*	Unidentified*	2
		*Peromatus*	*Peromatus* sp.	1
	**Reduviidae**	*Apiomerus*	*Apiomerus* sp.	1
		*Arilus*	*Arilus* cf. *carinatus*	1
		*Brontostoma*	*Brontostoma colossus*	4
			*Brontostoma discus*	3
			*Brontostoma haematodes*	1
			*Brontostoma rubrum*	3
			*Brontostoma* sp.	1
			cf. *Brontostoma* sp.	1
		*Cosmoclopius*	*Cosmoclopius* spp.	6
		*Eratyrus*	*Eratyrus mucronatus*	1
		*Heza*	*Heza* sp.	1
		*Graptocleptes*	*Graptocleptes bicolor*	2
		*Isocondylus*	*Isocondylus elongatus*	2
			cf. *Isocondylus elongatus*	1
		*Leogorrus*	*Leogorrus* sp.	1
		*Microtomus*	*Microtomus cinctipes*	2
		*Nitornus*	*Nitornus parkoi*	1
		*Oncocephalus*	*Oncocephalus* spp.	2
		*Opisthacidius*	*Opisthacidius* spp.	3
		*Panstrongylus*	*Panstrongylus geniculatus*	11
			*Panstrongylus lignarius*	2
			*Panstrongylus lutzi*	1
			*Panstrongylus* spp.	2
		cf. *Peregrinator*	cf. *Peregrinator* sp.	1
		*Pothea*	*Pothea* sp.	1
		*Rasahus*	*Rasahus hamatus*	4
			*Rasahu*s spp.	3
		*Rhiginia*	*Rhiginia* sp.	1
		*Rhodnius*	*Rhodnius* spp.	16
			*Rhodnius robustus*	108
			*Rhodnius pictipes*	4
			*Rhodnius* cf. *stali*	1
		*Rhyparoclopius*	*Rhyparoclopius* spp.	3
		*Salyavata*	*Salyavata* sp.	1
		*Sirthenea*	*Sirthenea* sp.	1
		*Sphaeridops*	*Sphaeridops pallescens*	1
			*Sphaeridops amoenus*	4
		*Stenopoda*	*Stenopoda* spp.	11
		*Triatoma*	*Triatoma brasiliensis brasiliensis*	2
		*Zelurus*	*Zelurus nigrolineatus*	1
			*Zelurus* sp.	1
		*Zelus*	*Zelus armillatus*	2
			*Zelus leucogrammus*	2
		Unidentified*	Unidentified*	10
	**Rhyparochromidae**	*Neopamera*	*Neopamera* sp.	1
	Unidentified*	Unidentified*	Unidentified*	3
Hymenoptera	Unidentified*	Unidentified*	Unidentified*	1
	**Pergidae**	Unidentified*	Unidentified*	1
Lepidoptera	**Esfingidae**	Unidentified*	Unidentified*	1
	**Geometridae**	Unidentified*	Unidentified*	1
	Unidentified*	Unidentified*	Unidentified*	1
Mantodea	**Mantidae**	*Stagmatoptera*	*Stagmatoptera binotata*	1
Orthoptera	**Tettigoniidae**	*Hexacentrus*	*Hexacentrus* sp.	1
Thysanoptera	Unidentified*	Unidentified*	Unidentified*	1
Unidentified*	Unidentified*	Unidentified*	Unidentified*	1
**TOTAL**				**465**

**TABLE 2:** Number of triatomines by species, sex, and habitat where the insect was found and reported to the “WhatsBarb” project.

Species	Unknown Sex	Males		Females		Total
		Indoor	Outdoor	Indoor	Outdoor	
*Eratyrus mucronatus*	0	0	0	0	1	1
*Panstrongylus* spp.	0	0	1	0	1	2
*Panstrongylus geniculatus*	1	5	3	2	0	11
*Panstrongylus lignarius*	0	0	1	1	0	2
*Panstrongylus lutzi*	0	0	0	1	0	1
*Rhodnius* spp.	8	5	0	3	0	16
*Rhodnius robustus*	3	32	4	64	5	108
*Rhodnius pictipes*	0	0	1	3	0	4
*Rhodnius* cf. *stali*	0	0	0	1	0	1
*Triatoma brasiliensis brasiliensis*	0	0	0	0	2	2
Unidentified (data loss)	2	0	0	0	0	2

**FIGURE 1:**
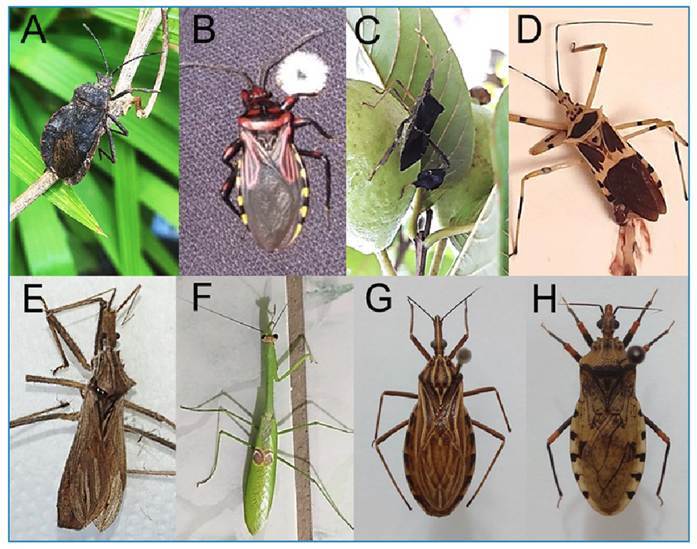
Main non-triatomine insects and kissing bugs recorded by the "WhatsBarb" volunteers between 2019 and 2024. **A:** *Spartocera* sp. (Coreidae: Coreinae)*,* **B:** *Brontostoma colossus* (Reduviidae: Ectrichodiinae)*,* **C:** *Leptoglossus* sp. (Coreidae: Coreinae)*,* **D:** *Zelus armillatus* (Reduviidae: Harpactorinae), **E:** *Stenopoda* sp. (Reduviidae: Stenopodainae), **F:** *Stagmatoptera binotata* (Mantidae: Vatinae), **G:** *Rhodnius robustus* (Reduviidae: Triatominae), **H:** *Panstrongylus geniculatus* (Reduviidae: Triatominae)*.*

Among the identified genera of insects usually confused with kissing bugs, the largest number of records was observed for *Spartocera*, *Leptoglossus* (Coreidae), *Brontostoma*, *Zelus*, and *Stenopoda* (Reduviidae) (Figure 1 A-E); Mantodea specimens were also recorded (Figure 1 F). For triatomines, there was a predominance of species belonging to the genera *Rhodnius* and *Panstrongylus* (Figure 1 G-H).

### Geographical Distribution of Insect Records

Initially, the project was created to meet community needs in the state of Rondônia; however, digital campaigns boosted the project’s visibility and expanded data and image gathering to a national scale, with contacts coming from 20 of the 26 states in Brazil, in addition to the Federal District. The volunteers collected more insect orders in Rondônia and Mato Grosso (Table 1).

### Challenges in Insect Diagnosis and Data Recording

Some challenges were noted in recording specimens, and systematizing and standardizing the information. Among these, low image resolution and lighting type were the main challenges identified. We created a digital campaign to guide volunteers to take photographs with adequate lighting, focusing on the angle and structure of the insect (mouthparts). As we did not measure the impact of this intervention on the quality of the new images, this task requires further investigation. In addition, because of fear of the insect being a kissing bug, they destroyed or crushed the specimen. To address this issue, collection pots with gloves were distributed to the local population, together with educational campaigns that explained on how to safely capture insects to avoid direct contact and prevent information loss and/or omission of insect encounters. However, despite these difficulties, it was possible to diagnose the images/videos sent by the project volunteers in almost all cases.

### Health Education Strategies

Four digital campaigns were conducted using WhatsBarb platform. Every year, we remember the project volunteers on World Chagas Disease Day, April 14, highlighting the importance of raising awareness about the fight against CD. "Intrusion of kissing bugs: know to prevent" is another campaign, which explains that triatomines are sylvatic insects in our region, that artificial lights attract them, as well as other insects, and that non-triatomines can bite to defend themselves, in addition to mentioning the importance of Citizen Surveillance. Basic orientations on care of yard, household waste, dwelling structure, location of artificial lights, care in recreational activities (forest tourism, hunting and fishing), and food safety were the themes of the campaign "Kissing bugs and Chagas disease: how to avoid". The campaigns proved to be effective, as we received contact from volunteers regarding the protective measures they were using, such as window screens and mosquito nets (distributed by the malaria prevention program). Using their photos, we developed the campaign "Citizen Surveillance in the fight against Chagas disease.”

## DISCUSSION

Currently, there is a new paradigm in the transmission of *Trypanosoma cruzi*, which causes CD, and the urbanization of triatomines, which, owing to their biological and behavioral characteristics, are prone to adaptation to urban environments. Some triatomine species are intrusive into households and even colonize urban areas, resulting in augmented encounter records in the last three decades^22^ ^-^ ^24^.

Therefore, alternative strategies for the prevention of CD are urgently required. The “WhatsBarb” platform has proven to be an effective interaction tool because of its global extent, rapid information flow, and public acceptance. In addition to its low cost, this tool allows for the systematization of insect and CD vector data, enabling both health promotion and inventories of local and regional biodiversity. 

This approach has been increasingly used as an information source in CD prevention programs, demonstrating its usefulness. In Mexico, a pilot community program obtained pictures and specimens of triatomines, which were then analyzed for *Trypanosoma cruzi* presence and bloodmeal sources. Forty-four triatomines were found in 15 states in Mexico and one triatomine was found in Nicaragua, totaling nine species. The pilot program also received contacts from Colombia, the United States, Peru, El Salvador, and Venezuela. *Trypanosoma cruzi* was detected in 8 of the 12 physical specimens, all of which were determined to be TcI by multiplex Real-Time PCR assays. The main sources of blood meals were humans, dogs, wood rats, doves, and anuran toads^25^.

In agreement with the One Health approach, WhatsBarb records can contribute to scientific knowledge and protection of insect biodiversity. The diversity and abundance of some insect groups have decreased, whereas other insect lineages have expanded their distribution range or increased their populations, including both aquatic and terrestrial insects^26^. However, despite the creation of various social media platforms and networks, tropical fauna remains underestimated, and many gaps still exist regarding the decline or stability of insect populations in this region^27^.

To explain the discrepancy in the number of contacts in February 2019, our database was examined, and local news articles published in digital media were consulted. The most plausible explanation is the wide divulgation of the first case of CD with potential vector transmission in the state of Rondônia, and other news presentations about the occurrence of beetles and vectors of CD in January 2019^28^ ^,^ ^29^. The WhatsBarb campaigns were launched during the same period. The use of the term “beetles” by the media, (a popular name for insects of the Coleoptera order) may have confounded the population and led them to a search for support in health and surveillance systems. Another explanation might be related to insect seasonality in the Amazon, where rainy seasons trigger an increase in insect populations (December, January, and February)^30^; however, this theory was discarded owing to a lack of monthly patterns.

Furthermore, some image records and specimens may have direct applications in taxonomic studies on Reduviidae. An insect provided by a project volunteer allowed scientific advancement in the status of *Sphaeridops pallescens* (Walker, 1873) and its revalidation, as it was previously considered a junior synonym of *Sphaeridops amoenus*. Hence, this contribution enabled the revision of the genus, redescription of the species, and updating of the taxonomic key^31^.

Currently, several digital tools have been developed for identifying triatomines, such as Triatodex^32^ and TriatoKey^33^. However, these tools require the user to install an application and/or understand the technical terms, which may reduce participant adherence. The WhatsBarb platform, which is based on the WhatsApp application, enables widespread communication without requiring participants to download additional applications to resolve their queries. In this sense, other strategies have been successful, such as the identification of triatomines using convolutional neural networks, based on photos taken by citizen scientists using cell phones^34^.

Even in the face of advances in digital and imaging technologies, the correct identification of kissing bug species by the population still requires the work of specialists in Entomology and Medical Entomology. This formal support has been provided for the identification of triatomines from photos, using absolute size measurements^35^, remote supervision during insect collection and identification by citizen scientists^11^, specialized taxonomic training for kissing bug identification through apps^32^, population responses, insect identification by biologists, and interventions (inspection and treatment with residual insecticide) by vector control experts^14^.

The main limitations of our study are the missing data on insects and the current geographic extension of the project. Because of image quality, loss of insect body parts, and limited volunteer records, the WhatsBarb database could not be further explored. The uneven coverage in WhatsBarb’s geographical representativeness resulted from the predominance of records from Rondônia and Mato Grosso, while states such as Amapá, Acre, and Sergipe were underrepresented. This indicates the need for intense campaigns in these regions to broaden the scope and impact of the project.

Thus, digital tools resulting from the combination of professional expertise and artificial intelligence could contribute to the shortage of taxonomists in surveillance systems, with the participation of society and presenting low cost, speed, and unlimited geographic scale^19^. However, training health and surveillance professionals to identify these insects is essential for the success of CD control programs, considering the importance of intervention actions on a local scale^36^.

## CONCLUSION

Despite some challenges regarding the quality of records (images and videos), the WhatsBarb database allowed us to measure encounters with both triatomine and non-triatomine insects, as well as to describe their occurrence on a national scale. Our study demonstrated that the frequency of non-triatomine insects was higher than expected, and approximately one-third of the volunteers were able to recognize insects as vectors of *Trypanosoma cruzi*. This quick, low-cost, dissemination-prone, and user-friendly interface strategy can and should be improved and will be of great use in surveillance systems. Our findings are encouraging and demonstrate the power of digital and social media for (i) disseminating digital health campaigns, (ii) recording and replying to volunteer questions on disease transmission, (iii) compiling biodiversity data, and (iv) identifying risk situations in remote locations relatively quickly, which could aid in mapping, preventing, and controlling CD vectors, and could be applied to other neglected diseases.
